# Effects of synchronous versus sequential scalp acupuncture and transcutaneous auricular vagus nerve stimulation on post-stroke cognitive impairment based on multimodal assessments: a single-center randomized controlled trial protocol

**DOI:** 10.3389/fneur.2026.1931213

**Published:** 2026-10-20

**Authors:** Jingyi Wei, Xihua Liu, Zhipeng Pan, Chen Wei, Ran Wang, Xiaojing Wang, Sai Ma

**Affiliations:** 1 College of Rehabilitation Medicine, Shandong University of Traditional Chinese Medicine, Jinan, China; 2 Department of Rehabilitation, Affiliated Hospital of Shandong University of Traditional Chinese Medicine, Jinan, China

**Keywords:** multi-modal fusion, post-stroke cognitive impairment, scalp acupuncture, study protocol, transcutaneous auricular vagus nerve stimulation

## Abstract

**Background:**

Post-stroke cognitive impairment (PSCI) significantly burdens individuals and society. While scalp acupuncture and transcutaneous auricular vagus nerve stimulation (taVNS) show promise for alleviating cognitive deficits, the optimal temporal sequence of their combined delivery remains uncertain. This study aims to evaluate the efficacy and safety of synchronous versus sequential delivery of scalp acupuncture and taVNS for PSCI, and explore central-peripheral neuromodulatory responses associated with intervention timing via multimodal assessment.

**Methods:**

This prospective, randomized controlled trial will allocate 96 eligible PSCI patients equally into three groups. All receive conventional treatment alongside scalp acupuncture and taVNS. The synchronous group undergoes concurrent acupuncture and taVNS. Control Group 1 receives acupuncture followed by taVNS, while Control Group 2 receives taVNS followed by acupuncture. Treatments are administered once daily, 5 days per week, for 4 weeks. To assess the impact of temporal sequencing, multimodal data will be compared at baseline, week 4, and week 8 follow-up. Assessments encompass cognitive function (MoCA, MMSE), activities of daily living (MBI), neuroimaging (rs-fMRI), neuroelectrophysiology (ERP-P300), cerebral hemodynamics (TCD), and adverse events.

**Discussion:**

Results are expected to evaluate the preliminary efficacy and safety of synchronous versus sequential scalp acupuncture-taVNS delivery for PSCI. Furthermore, findings may help explore the temporal dynamics of neuromodulation across brain networks, neural electrical activity, and cerebral hemodynamics, contributing to the evidence base for standardizing integrative Chinese-western cognitive rehabilitation protocols.

**Clinical trial registration:**

https://www.chictr.org.cn/, Identifier [ChiCTR2600125558].

## 1 Introduction

Post-stroke cognitive impairment (PSCI) is one of the most common disabling complications following stroke (1). Global epidemiological data indicate that the incidence of cognitive impairment within 3–6 months after stroke can reach 40%–60%, and approximately one-third of patients progress to vascular dementia within several years (2, 3). PSCI not only severely impedes the recovery of neurological function and activities of daily living, but also significantly elevates the risk of stroke recurrence and all-cause mortality, imposing heavy caregiving and economic burdens on families and society (4, 5). With the accelerated aging of the population and the continued rise in stroke incidence in China, PSCI has become a critical clinical challenge in neurorehabilitation that urgently needs to be addressed.

Currently, therapeutic strategies for PSCI predominantly rely on cognitive rehabilitation training and pharmacological interventions (6, 7). However, pharmacotherapy (e.g., donepezil, memantine) has limited efficacy and may be accompanied by adverse effects, whereas conventional cognitive rehabilitation training, although yielding some benefits, is slow to take effect and exhibits considerable inter-individual variability (8, 9). Consequently, exploring safe, effective, and scalable non-pharmacological intervention strategies has become a research hotspot and an urgent clinical need in the field of PSCI rehabilitation.

The “central-peripheral-central” closed-loop rehabilitation theory has attracted increasing attention in recent years. Rather than denoting an engineering-based adaptive system driven by real-time physiological feedback, this theory used in this study serves as a conceptual neurorehabilitation framework that emphasizes the temporal coordination of central interventions (e.g., transcranial magnetic stimulation, scalp acupuncture) and peripheral interventions (e.g., peripheral nerve electrical stimulation, motor training) (10), potentially optimizing neural functional recovery through a closed-loop mechanism of “central activation-peripheral input-central remodeling” (11). However, the translational application of this theory in the domain of cognitive rehabilitation still lacks high-level clinical evidence.

Scalp acupuncture is a distinctive technique within the system of traditional Chinese medicine acupuncture, designed specifically for brain disorders. Based on the functional localization of the cerebral cortex and meridian-acupoint theory, it stimulates corresponding functional projection areas on the scalp, thereby directly modulating cortical excitability, improving regional cerebral perfusion, and promoting neurogenesis and synaptic plasticity (12). Given that its primary therapeutic targets are the cerebral cortex and intracerebral functional networks, scalp acupuncture is categorized as a central neuromodulatory intervention in this framework, acting via direct “top-down” regulation of central nervous system activity. This distinguishes it from peripheral interventions such as taVNS, which exert central effects indirectly via afferent nerve pathways. Multiple clinical studies have confirmed that scalp acupuncture can effectively improve attention, executive function, and episodic memory in PSCI patients, with a low incidence of adverse events and good clinical tolerability (13).

In recent years, non-invasive neuromodulation techniques have shown broad promise in neurorehabilitation. Transcutaneous auricular vagus nerve stimulation (taVNS), as an emerging non-invasive neuromodulation approach, stimulates the auricular branch of the vagus nerve, activating vagal afferent projection pathways to the central nervous system, and thereby modulating central pathways involved in neuroplasticity, anti-inflammatory processes, angiogenesis, and blood–brain barrier protection (14, 15). Existing studies have demonstrated preliminary efficacy of taVNS in improving post-stroke motor, sensory, cognitive, and neuropsychiatric disorders. However, clinical research on taVNS for PSCI remains relatively limited, and questions regarding optimal stimulation parameters and temporal combination paradigms with other therapies have yet to be systematically explored (16).

Although scalp acupuncture and taVNS each exert cognitive improvement effects from the perspectives of central regulation and peripheral nerve stimulation, few studies have explored the clinical efficacy of their combined application. Current research is still constrained by three key scientific gaps. First, the impact of combined application and intervention timing on treatment efficacy has not been systematically investigated; it remains unclear whether synchronous and sequential delivery of scalp acupuncture and taVNS are associated with different clinical outcomes, directly impeding the optimization of clinical treatment protocols. Second, existing studies predominantly use neuropsychological scales as primary outcome measures, lacking multidimensional objective evidence from neuroimaging, neuroelectrophysiology, and cerebral hemodynamics, which makes it difficult to explore neurophysiological response patterns associated with different intervention timings at the levels of brain networks, neural electrical activity, and hemodynamics (17). Third, an efficacy evaluation system based on the fusion of multisource heterogeneous data has yet to be established, limiting the characterization of multimodal response patterns and the identification of features potentially associated with cognitive improvement.

Building upon this rationale, the present study adopts a prospective, randomized, controlled trial design to systematically compare the effects of scalp acupuncture and taVNS delivered in synchronous versus sequential modes on cognitive function, activities of daily living, and multimodal neurophysiological measures in patients with PSCI, aiming to evaluate whether clinical outcomes differ according to intervention timing and to exploratorily characterize neurophysiological response patterns associated with synchronous and sequential delivery. By utilizing sequential active interventions as control arms rather than a conventional sham group, this study design is specifically tailored to isolate and investigate the temporal dynamics of central-peripheral neuromodulation. Furthermore, by leveraging feature-level fusion strategies and machine learning, multidimensional data will be integrated on an exploratory basis to identify candidate multimodal features associated with cognitive improvement and intervention timing. This work is expected to provide preliminary evidence for the temporal optimization of the “central-peripheral-central” closed-loop rehabilitation theory and ultimately contribute to a standardized, generalizable integrative Chinese-Western medicine rehabilitation protocol for PSCI.

The implementation of this study is expected to offer a safe and promising non-pharmacological rehabilitation strategy for PSCI patients, while enriching and refining the “central-peripheral-central” closed-loop rehabilitation theoretical framework. It may offer clinical insights for the integration of Chinese-Western medicine rehabilitation and support the transition in post-stroke cognitive rehabilitation towards more optimized multimodal strategies, and lay a methodological foundation for subsequent multicenter, large-sample clinical trials.

## 2 Materials and methods

### 2.1 Study design

This is a prospective, open-label, single-center, randomized controlled trial. The study will include 96 post-stroke patients with cognitive impairment, divided into three groups, with 32 individuals in each group. All participants will receive standard routine care, including management of underlying diseases, neurological rehabilitation training, and standardized cognitive rehabilitation training. On this basis, participants in Control Group 1 will receive scalp acupuncture followed by taVNS, while those in Control Group 2 will receive taVNS prior to scalp acupuncture; an interval of at least 3 h will be maintained between the two interventions in both sequential groups. For the synchronous group, taVNS will be delivered simultaneously during the scalp acupuncture needle retention period. The intervention schedule for all three groups is identical: once daily, 5 days per week, for a total treatment course of 4 weeks.

The study was approved by the Ethics Committee of the Affiliated Hospital of Shandong University of Traditional Chinese Medicine (Approval No: 2026-102-YJS) and prospectively registered with the Chinese Clinical Trial Registry (Identifier: ChiCTR2600125558) on May 28, 2026. Written informed consent will be obtained from all participants prior to their enrollment in the trial. This trial will be conducted in accordance with the SPIRIT (Standard Protocol Items: Recommendations for Interventional Trials) checklist (Supplementary material S1). The trial flow is presented in Figure 1, and the timeline for participant enrollment, interventions, and outcome assessments is shown in Table 1.

**Figure 1:**
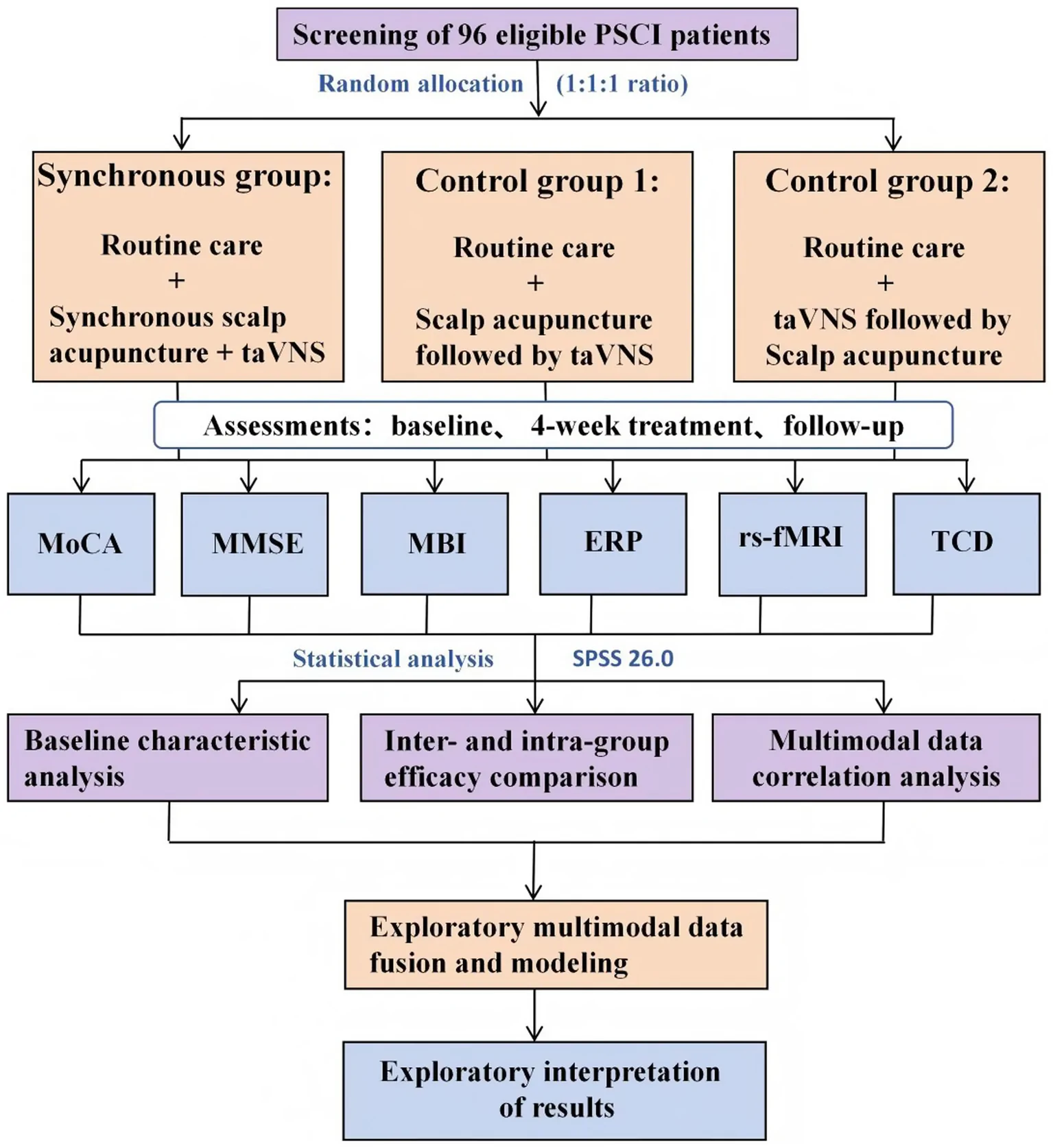
Study flowchart. MoCA, Montreal Cognitive Assessment; MMSE, Mini-Mental State Examination; MBI, Modified Barthel Index; ERP, Event-related potentials; rs-fMRI, Resting-state functional MRI; TCD, Transcranial Doppler Ultrasonography.

**Table 1:** Study procedure timeline.

	Screening	Baseline	Treatment period		Followed-up period
	Week −1	Week 0	Week 1–3	Week 4 (End of Treatment)	Week 8
Screening and recruitment					
Inclusion and exclusion	√				
Sign the informed consent	√				
Randomization		√			
Interventions					
Synchronous group			√	√	
Control group1			√	√	
Control group 2			√	√	
Assessments					
MoCA		√		√	√
MMSE		√		√	√
MBI		√		√	√
ERP		√		√	√
rs-fMRI		√		√	√
TCD		√		√	√
Others					
Adverse events			√	√	√
Multimodal data fusion				√	

Week −1, screening period; Week 0, baseline evaluation; Week 1–3, treatment period; Week 4, end of treatment; Week 8, follow-up period. MoCA, Montreal Cognitive Assessment; MMSE, Mini-Mental State Examination; MBI, Modified Barthel Index; ERP, Event-Related Potential; rs-fMRI, Resting-State Functional Magnetic Resonance Imaging; TCD, Transcranial Doppler Ultrasonography.

### 2.2 Participants

#### 2.2.1 Diagnostic criteria

The diagnosis of stroke will be established in accordance with the diagnostic criteria for cerebral hemorrhage or cerebral infarction specified in the Diagnostic criteria of cerebrovascular diseases in China (version 2019) (18), and confirmed by cranial CT or MRI imaging examinations. The diagnosis of cognitive impairment will follow the diagnostic criteria for post-stroke cognitive impairment outlined in the *Expert Consensus on the Management of Post-Stroke Cognitive Impairment* (2021) (19).

#### 2.2.2 Inclusion criteria

Eligible participants must meet all of the following criteria simultaneously: (1) first-ever stroke patients meeting the above diagnostic criteria, aged 30–80 years old, within 1 to 6 months post-stroke onset, with Chinese as their native language; (2) cognitive impairment confirmed by the Mini-Mental State Examination (MMSE) and Montreal Cognitive Assessment (MoCA) (20, 21), meeting either of the following criteria: ① MoCA score < 26 points (an additional 1 point is added for participants with ≤12 years of education), or ② MMSE score ≤17 for illiterate patients (no formal education), ≤20 for primary school education (≤6 years of education), or ≤24 for secondary education or above (>6 years of education); (3) in the recovery phase of stroke, with stable vital signs, clear consciousness, adequate comprehension ability, no severe aphasia, and able to cooperate with all assessments and treatments; (4) no use or injection of medications affecting cognitive function (e.g., donepezil, memantine, huperzine A) within 1 month prior to enrollment; (5) no visual or auditory impairment, and no strabismus; and (6) written informed consent obtained from the patient and/or their family members.

#### 2.2.3 Exclusion criteria

Patients will be excluded if they meet any of the following criteria: (1) severe carotid artery stenosis (>50%) or unstable plaques confirmed by routine ultrasound; skin lesions, inflammation, scars, or other abnormalities at the stimulation sites for taVNS and scalp acupuncture; (2) presence of implanted devices such as pacemakers or cochlear implants, or skull defects; (3) previous vagus nerve surgery or presence of intracranial metal foreign bodies; (4) a history of acupuncture syncope or blood syncope, or contraindications to acupuncture therapy such as hematological diseases or coagulation disorders; (5) pre-existing significant cognitive impairment prior to stroke onset, or cognitive impairment caused by other neurodegenerative diseases (e.g., Alzheimer’s disease [AD]), demyelinating diseases, or metabolic abnormalities; (6) use of central nervous system (CNS)-acting medications (e.g., sedative-hypnotics, antiepileptics, antipsychotics) within 1 month prior to enrollment; (7) history of epilepsy, family history of psychiatric disorders, history of drug abuse, drug dependence, or alcohol dependence; (8) severe insufficiency of vital organs (e.g., heart, liver, kidney) or malignant tumors; and (9) current participation in another clinical trial.

### 2.3 Recruitment process

Participants will be recruited from the Department of Rehabilitation of the Affiliated Hospital of Shandong University of Traditional Chinese Medicine, which mainly improves various functional impairments after stroke through integrated traditional Chinese and Western medicine rehabilitation. Recruitment methods include distributing brochures in the hospital and publishing information on the WeChat public platform. Post-stroke patients who meet the eligibility criteria will become the subjects of this study. The research team will provide professional treatment to participants. Scalp acupuncture, taVNS, instrument-based examinations, and scale assessments will be provided free of charge. However, pharmacotherapy and conventional rehabilitation treatment are not covered within the scope of free services. Interested patients who wish to participate in this trial can contact the researchers by phone or WeChat. We encourage patients to ask as many questions as possible to the researchers before deciding to participate until they fully understand the content, risks, and benefits of the trial. Detailed information such as gender, age, disease duration, stroke type, paretic side, Brunnstrom stage and MoCA score will be recorded by the researchers in the respective case report forms at admission. During the treatment period, researchers will collect and monitor data including scale assessment reports, neuroimaging indicators, neuroelectrophysiological indicators, and cerebral hemodynamic parameters of all participants.

### 2.4 Sample size

The change in the MoCA score from baseline to week 4 was designated as the primary outcome. Based on previous literature (22, 23) and pilot data, we hypothesized the expected mean changes in MoCA scores to be 4.9 points for the synchronous group, and 1.5 points for both control group 1 and control group 2. This 3.4-point between-group difference exceeds the minimal clinically important difference (MCID) of 2–3 points for MoCA in neurological populations (23), supporting the clinical relevance of the hypothesized effect. With a pooled standard deviation of 4.2, the standard deviation of these expected population means ( $\sigma_{m}$ ) is calculated to be approximately 1.60. According to the formula for a one-way analysis of variance ( $f=\sigma_{m}/\sigma$ ), this yields an effect size of Cohen’s f≈0.38 .

Sample size was estimated using the One-Way ANOVA module of PASS 15 software. With a two-sided significance level of α=0.05 , a statistical power of 1−β=0.90 , and a 1:1:1 randomization ratio, an effect size of f=0.38 required 29 participants per group. Assuming an approximately 10% attrition rate, 32 participants per group were planned to ensure at least 29 completers per group, resulting in a total sample size of 96 participants. This attrition estimate is conservative and consistent with conventional rates reported in short-term single-center rehabilitation trials with multimodal assessments. The one-way ANOVA framework was used solely for sample-size estimation based on the anticipated between-group differences in the primary MoCA endpoint. The prespecified primary confirmatory analysis of the primary outcome will be performed using the MMRM described in Section 2.10.1. The two prespecified comparisons between the synchronous group and each sequential control group at week 4 will be evaluated using MMRM-derived contrasts with Holm adjustment for multiple comparisons. The trial is powered specifically for the MoCA clinical endpoint; the multimodal neuroimaging and machine-learning analyses are exploratory and hypothesis-generating.

### 2.5 Randomization, allocation concealment, and blinding

Three random number sequences will be generated using IBM SPSS Statistics version 26.0 (IBM Corp., Armonk, NY, USA). Eligible patients with PSCI will be assigned consecutive sequential numbers in the order of enrollment. A total of 96 eligible PSCI patients will be randomly allocated to three groups at a 1:1:1 ratio, with 32 patients in each group, stratified by stroke type (ischemic versus hemorrhagic). The personnel responsible for generating the randomization sequence will not be involved in patient recruitment, intervention administration, efficacy assessment, data collection, or statistical analysis.

For allocation concealment, group assignment results will be sealed in sequentially numbered, opaque envelopes. Patients will be enrolled strictly following their sequential enrollment numbers, and the corresponding envelopes will be delivered to the therapists administering the study interventions. The allocation scheme will be unsealed and executed by the treating therapist only after the patient has been formally enrolled in the trial.

Owing to the inherent characteristics of the interventions, blinding of participants and acupuncturists will not be feasible. However, to minimize potential bias, outcome assessors, imaging data collectors, and statisticians will remain blinded to group assignment throughout the trial. These personnel will be unaware of patients’ allocation status and will not take part in any therapeutic intervention procedures.

### 2.6 Intervention

All participants will receive standard routine care, including management of underlying diseases, neurological rehabilitation training, and standardized cognitive training. On this basis, participants in Control Group 1 will receive scalp acupuncture followed by taVNS, while those in Control Group 2 will receive taVNS prior to scalp acupuncture, with an interval of at least 3 h between the two interventions. For the synchronous group, taVNS will be administered concurrently during the scalp acupuncture needle retention period. All interventions will be performed once daily, 5 days per week, for a total treatment course of 4 weeks.

#### 2.6.1 Conventional baseline treatment

All participants will receive standardized comprehensive treatment in accordance with the *Expert Consensus on the Management of Post-Stroke Cognitive Impairment* (2021) (19).

*Management of underlying diseases*: Symptomatic management including blood glucose and blood pressure control, lipid-lowering therapy, and atherosclerotic plaque stabilization will be provided. On the premise of not violating clinical principles, medications that alter cortical excitability (e.g., sedatives and antidepressants) will be avoided. Participants requiring such medications for clinical necessity will discontinue the assigned study intervention but will be strongly encouraged to complete all scheduled outcome assessments to remain in the Intention-to-Treat (ITT) population, unless they explicitly withdraw consent.

*Neurological rehabilitation training*: The comprehensive rehabilitation program consists of therapeutic exercise (including neurofacilitation training for the hemiplegic limb, trunk control training, and muscle strengthening), occupational therapy (including motor control training for the hemiplegic upper limb and hand, scapular control training, and activities of daily living training), and physical agent modalities (such as paraffin therapy and neuromuscular electrical stimulation). The regimen will be individualized according to each patient’s functional status and delivered by the attending therapist. Treatment will be provided once daily, 5 days per week, for four consecutive weeks.

*Cognitive training*: Prior to treatment, rehabilitation physicians will assess each patient using MMSE and MoCA. Based on the assessment results, cognitive deficits will be identified and an individualized training program will be developed, covering attention, orientation, memory, calculation, and executive function. Each session lasts 30 min, once daily, 5 days per week, for 4 weeks, and patient rehabilitation progress will be monitored throughout the intervention period.

#### 2.6.2 Transcutaneous auricular vagus nerve stimulation

The Rishen’an tVNS510 auricular vagus nerve stimulator (Changzhou Rishen’an Medical Device Co., Ltd., Jiangsu, China) will be used to deliver taVNS. Prior to each session, the electrode and the skin of the left auricular concha, a primary cutaneous area innervated by the auricular branch of the vagus nerve, will be cleaned with cotton balls soaked in 75% alcohol to remove excess sebum and ensure optimal electrical conductivity. A single earplug electrode will then be placed in the left concha; no electrode is applied to the right ear. After turning on the device, the working mode is selected via the control button. Stimulation parameters will be set as follows: waveform, continuous; frequency, 25 Hz; pulse width, 200 μs; and current amplitude ranging from 1.0 to 5.0 mA, customized daily to a level just below the participant’s individual pain threshold. Each stimulation session lasts 30 min and is administered once daily, 5 days per week, for a total of 4 weeks.

#### 2.6.3 Scalp acupuncture

The acupoints selected are Baihui (GV20), Shenting (GV24), and Sishencong (EX-HN1). With the patient seated, the skin over the acupoints will be routinely disinfected with 75% alcohol cotton balls. The practitioner will use 0.35 mm × 40 mm disposable Hwato acupuncture needles, inserting them obliquely into the scalp at a 15° to 30° angle to a depth of 20 to 25 mm until the subgaleal space is reached. The needles will be retained for 30 min per session; during retention, twirling manipulation will be applied for 2 min every 10 min at a frequency of 150 twists per minute. The presence of de qi (a sensation of soreness, numbness, distension, or heaviness) will be required and recorded for each session. Treatment will be administered once daily, 5 days per week, for a total of 4 weeks. The scalp acupuncture points are shown in Figure 2.

**Figure 2:**
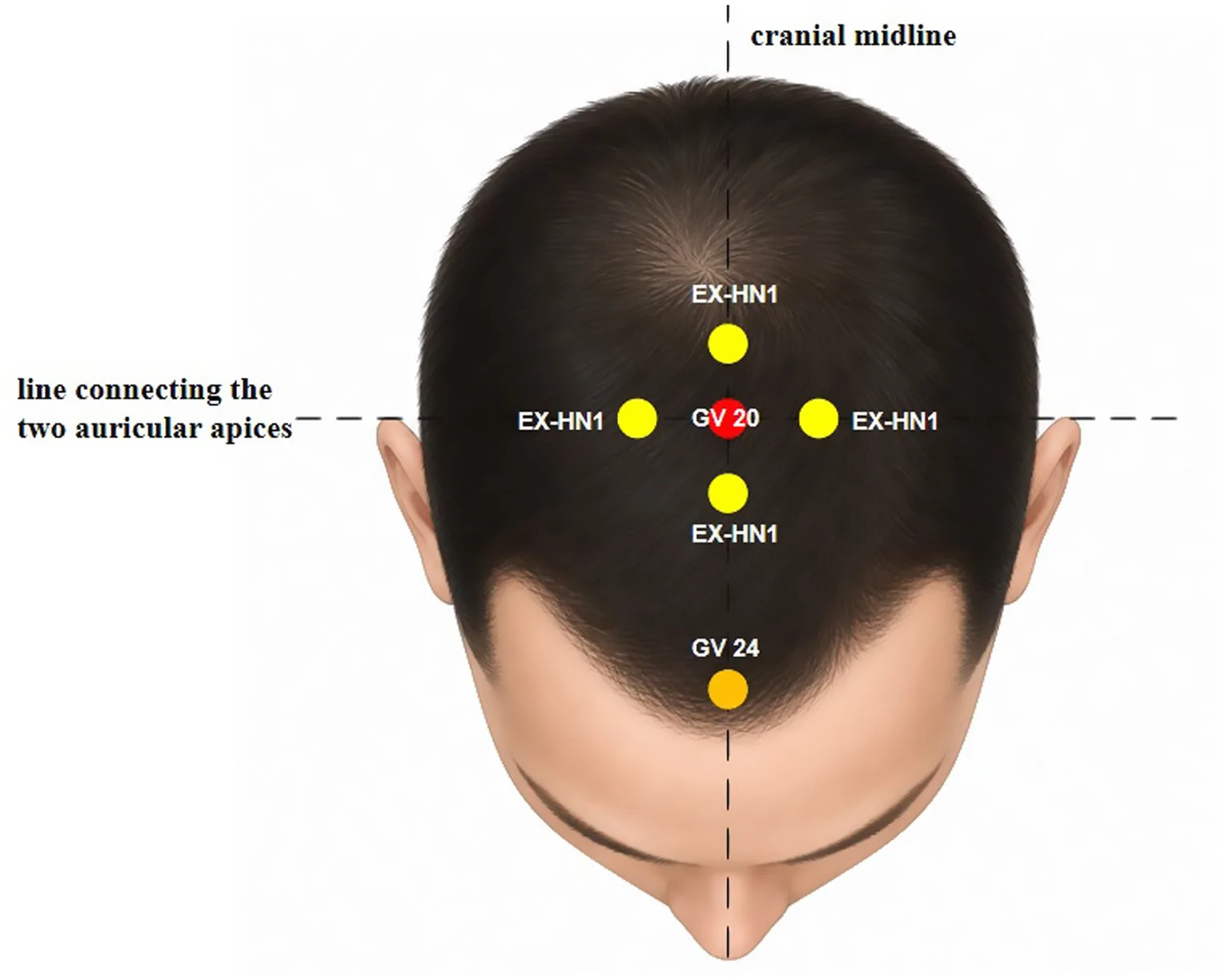
Scalp acupuncture points (superior view). The central point indicates Baihui (GV 20); the four surrounding points represent the extra cranial acupoint Sishencong (EX-HN 1); the anterior frontal point denotes Shenting (GV 24).

#### 2.6.4 Intervention fidelity

To ensure high intervention fidelity and consistency across all groups, all scalp acupuncture and taVNS procedures will be performed by a dedicated team of three certified acupuncturists and three rehabilitation therapists with at least 5 years of clinical experience. Prior to trial initiation, all practitioners will undergo standardized training specifically focused on this study protocol, encompassing precise acupoint localization, standardized taVNS parameter settings, and exact timing control for the synchronous/sequential administrations. Furthermore, an independent clinical monitor will conduct random on-site fidelity checks on approximately 10% of the treatment sessions to ensure strict adherence to the intervention protocols.

### 2.7 Outcome measurements

#### 2.7.1 Primary outcome

In this trial, the primary outcome will be defined as the change in MoCA scores from baseline to week 4 of treatment. The assessment covers eight cognitive domains, including visuospatial skills, executive function, memory, language, attention and concentration, calculation, abstract thinking, and orientation. The total score is 30 points, with ≥26 points indicating normal, 18–25 points indicating mild cognitive impairment, 10–17 points indicating moderate cognitive impairment, and <10 points indicating severe cognitive impairment. For mild cognitive impairment, MoCA is more sensitive in screening (24). To mitigate potential practice effects from repeated testing, alternate validated equivalent Chinese versions of the MoCA will be utilized across the assessment time points.

#### 2.7.2 Secondary outcomes

##### 2.7.2.1 Mini-mental state examination (MMSE)

MMSE, originally developed by Folstein et al. (25), has become a widely adopted cognitive function assessment tool worldwide. The scale comprises the following items with corresponding scores: 5 points for orientation to time, 5 points for orientation to place, 3 points for immediate memory, 5 points for attention and calculation, 3 points for delayed recall, 2 points for naming, 1 point for verbal repetition, 1 point for reading ability, 3 points for three-step command execution, 1 point for writing ability, and 1 point for constructional ability. The maximum total score is 30, with lower scores indicating more severe cognitive impairment. Cut-off scores for identifying cognitive impairment are stratified by educational attainment: ≤17 points for illiterate individuals with no formal education, ≤20 points for those with primary school education (≤6 years of schooling), and ≤24 points for those with secondary school education or above (>6 years of schooling). This scale focuses on core cognitive domains including orientation and memory, and demonstrates high sensitivity for moderate to severe cognitive impairment (25, 26).

##### 2.7.2.2 Modified Barthel index (MBI)

MBI is a globally recognized quantitative instrument for evaluating activities of daily living (ADL). It comprises 10 items: bowel control, bladder control, dressing, toileting, feeding, bathing, grooming, bed-to-chair transfer, ambulation, and stair climbing. Each item is rated across four tiers based on whether assistance is required and the degree of support needed. Higher total scores indicate less dependence on assistance, while lower scores reflect a greater degree of functional dependency. The maximum total score is 100. Specifically, scores of 0–20 denote extremely severe functional impairment; 21–45 denote severe functional impairment; 46–70 denote moderate functional impairment; 71–99 denote mild functional impairment; and a score of 100 represents complete independence in ADL (25).

##### 2.7.2.3 Resting-state functional magnetic resonance imaging(rs-fMRI)

The GE Discovery MR 750 scanner (General Electric Company, USA) equipped with a 32-channel head scanning coil will be used. Prior to scanning, participants will be screened to ensure the removal of all ferromagnetic objects to prevent artifact interference. Participants will be positioned supine on the scanner bed, and the head will be fixed with a sponge pad to minimize head movement. White noise will be played through headphones to isolate external interference, and the patient will be allowed to close his eyes, stay awake, avoid thinking activities and body movement, and rest quietly for about 8 min. The scanning parameters will be set as follows: using Gradient Echo-Planar Imaging (GRE-EPI) sequence, Repetition Time (TR) = 2000 ms, Echo Time (TE) = 30 ms, Flip Angle = 90°, Field of View (FOV) = 220 mm × 220 mm, Matrix = 64 × 64, Slice Thickness = 3 mm, and a total of 240 time points will be collected. After scanning, the images will be preprocessed using SPM12 software. Preprocessing will be rigorously prespecified, including discarding the first 10 volumes, slice-timing correction, realignment (excluding participants with head motion > 2.0 mm or 2.0° rotation, and mean framewise displacement > 0.2 mm), spatial normalization, and smoothing (6-mm FWHM kernel). Nuisance variable regression will include the Friston-24 motion parameters, white matter, and cerebrospinal fluid signals, without global signal regression. Voxel-wise multiple comparisons will be corrected using the Gaussian Random Field (GRF) theory. For Region of Interest (ROI) analysis, the brain will be parcellated based on the Automated Anatomical Labeling (AAL) atlas. Finally, the time series of each brain region will be extracted and averaged. Correlation analysis will be performed between these averaged time series and every voxel in the whole brain to obtain correlation coefficients. These coefficients will be converted to normally distributed Z-values via Fisher’s z-transformation for functional connectivity analysis (27, 28). Functional connectivity, ALFF, and ReHo analyses are defined *a priori* as exploratory mechanistic endpoints rather than primary confirmatory outcomes.

##### 2.7.2.4 Event-related potentials (ERP)-P300

(I) Equipment

The Neuracle Pony NSM2 event-related potential system (Neuracle Technology Co., Ltd., Changzhou, China) and its accompanying data acquisition workstation will be employed for electrophysiological recording.

(II) Scalp electrode placement

Electrode positioning will adhere strictly to the International 10–20 system of electrode placement, defined by two anatomical reference lines. The first is the nasion–inion midline, along which five midline sites are marked: Fpz, Fz, Cz, Pz, and Oz. The second is the interaural line connecting the bilateral external auditory meati, along which five sites are marked: T3, C3, Cz, C4, and T4, with Cz serving as the intersection of the two lines. The Cz electrode will act as the active recording electrode, and the mid-frontal site Fz will serve as the reference electrode.

(III) Procedure

Prior to the P300 recording, participants will receive standardized instructions regarding the test purpose, experimental protocol, and required compliance. Pure-tone hearing screening will be conducted first, with hearing thresholds within 20 dB hearing level (HL) defined as eligible. A practice block will be administered before formal recording: two sets of pure tones with distinct frequencies but equal sound intensity will be delivered binaurally via headphones, corresponding to target and non-target stimuli, respectively. Participants will be instructed to silently count the high-frequency target tones (29).

All recordings will be performed in a sound-attenuated, electromagnetically shielded room. Participants will be seated in a comfortable position and instructed to remain awake, alert, and motionless throughout the session to minimize artifacts. The examiner will prepare the scalp with abrasive paste to reduce cutaneous impedance, followed by the application of conductive electrode gel. The black disc-shaped active electrode will be fixed at the vertex (Cz), the red disc-shaped reference electrode at the mid-frontal site (Fz), and the ground electrode at a site 5 cm proximal to the volar wrist crease on the tested side. Inter-electrode impedance will be maintained below 5 kΩ for all electrodes (30).

Short auditory stimuli will be presented in a pseudorandom order, with target stimuli (2,000 Hz) comprising 20% and non-target stimuli (1,000 Hz) 80% of all trials. Stimuli will be presented at 75 dB with a stimulus duration of 200 ms and an inter-stimulus interval of 1,000–1,500 ms. The EEG signals will be continuously sampled at 1000 Hz. A total of 200 trials will be recorded, including 40 target trials and 160 non-target trials. Participants will be required to count the target tones silently. Data will be filtered online (0.1–30 Hz), epoched from −200 to 800 ms, and baseline-corrected (−200 to 0 ms). Trials with artifacts exceeding ±80 μV will be rejected. A minimum of 30 valid target trials was retained for subsequent P300 analysis to ensure data reliability. P300 peak amplitude and latency will be extracted from a predefined 250–500 ms window.

##### 2.7.2.5 Transcranial Doppler ultrasonography (TCD)

(I) Equipment

A transcranial Doppler (TCD) blood flow analyzer (Beijing Yueqi Chuangtong Technology Co., Ltd., Beijing, China) with a 2.0 MHz probe will be utilized for the examination.

(II) Procedure

Patients will lie in the supine position on the examination couch and be instructed to remain quiet throughout the intracranial hemodynamic assessment. An appropriate amount of ultrasonic coupling gel will be applied to the temporal acoustic window. The probe will be placed horizontally on the patient’s temporal region and oriented toward the contralateral side, and blood flow directed toward the probe will be detected at a depth ranging from 40 mm to 65 mm. Concurrently, pressure will be applied to the anterior border of the sternocleidomastoid muscle to compress the common carotid artery, and a marked reduction in blood flow velocity will be observed to confirm vessel localization (31). This brief carotid compression (<3 s) will only be performed gently when strictly necessary for vessel identification. Patients with severe carotid stenosis (>50%) or unstable plaques are excluded to ensure the safety of this maneuver.

### 2.8 Adverse events

#### 2.8.1 Adverse event grading

Routine vital sign monitoring: body temperature, blood pressure, heart rate, and respiratory rate will be measured at designated assessment time points.

Adverse events (AEs) occurring during taVNS and scalp acupuncture interventions will be closely monitored and graded by severity as follows:

Adverse events (AEs) will be graded based on severity: Mild (transient, easily tolerated, requiring no treatment), Moderate (causes discomfort, interferes with usual activities, requires symptomatic treatment), and Severe (incapacitating, requires significant medical intervention or trial discontinuation). The expectedness, seriousness, and relationship to scalp acupuncture (e.g., hematoma, syncope) or taVNS (e.g., skin irritation) will be systematically documented, alongside actions taken and clinical outcomes.

#### 2.8.2 Adverse event management

Scalp acupuncture may cause adverse events including syncope, stuck needle, broken needle, hematoma, and subcutaneous hemorrhage. TaVNS may lead to local skin irritation, headache, and bradycardia. Should any adverse event occur during the study period, physicians will promptly administer standardized symptomatic management according to the nature and severity of the event. Throughout the trial, all untoward medical occurrences will be systematically and accurately documented, including the time of onset, duration, corrective interventions undertaken, and final clinical outcomes. All adverse events must be recorded in detail on the CRFs. In the event of any serious adverse event, it must be reported immediately to the Institutional Review Board/Ethics Committee, and the affected participant will be withdrawn from the trial without delay.

### 2.9 Data collection, management, and monitoring

#### 2.9.1 Data collection

All source data from the subjects, including scale scores and instrument-based assessment results, will be fully collected and recorded in the case report forms (CRFs) in an accurate, legible, complete, and standardized manner. Every field in the CRFs must be completed. If a correction becomes necessary, the original entry should remain legible and be crossed out with a single line; the correction must be accompanied by the date, the reason for the change, and the signature of the authorized person making the correction. To standardize concomitant exposure, a specific log will record routine rehabilitation treatment minutes, total sessions completed, missed sessions, and relevant concomitant medications. These variables will be monitored and compared across groups.

#### 2.9.2 Data management

All study data, including documents, assessment reports, and clinical trial results, will be systematically archived at the hospital in a manner that permits rapid retrieval. The archive will be managed by a designated administrator, and access will be restricted exclusively to authorized personnel. In addition, paper-based records will be digitized by scanning and stored in an encrypted electronic format.

#### 2.9.3 Data monitoring

Monitors will oversee whether the experiment is conducted in accordance with the study protocol and relevant regulations, whether there are errors or omissions in the original data, whether all CRFs are complete and accurate, and whether there are deviations from the original data.

#### 2.9.4 Protocol amendments

Any significant modifications to the study protocol (e.g., changes to eligibility criteria, outcomes, or statistical analysis plans) will require formal approval from the Ethics Committee prior to implementation. The clinical trial registry (ChiCTR) will be updated accordingly to ensure transparency.

#### 2.9.5 Dissemination policy

The findings of this trial will be submitted for publication in peer-reviewed scientific journals, regardless of the magnitude or direction of the effect. Results will also be disseminated at relevant national and international academic conferences to ensure the findings reach the scientific and clinical communities.

### 2.10 Statistical analyses

#### 2.10.1 Statistical analysis

All clinical data will be collected using standardized CRFs and stored in a secure, centralized database under the supervision of designated research personnel. Descriptive statistics and general statistical analyses will be conducted using IBM SPSS Statistics version 26.0 (IBM Corp., Armonk, NY, USA). Mixed-effects models for repeated measures (MMRM) will be fitted using the lme4 or nlme package in R version 4.3.0 or later, and multiple imputation will be performed using the mice package in R.

The primary efficacy analysis will follow the intention-to-treat (ITT) principle, whereby all randomized participants will be analyzed according to their originally assigned groups regardless of treatment adherence or trial completion. The MMRM will serve as the prespecified primary confirmatory analysis for the primary outcome. Post-baseline MoCA scores at week 4 and week 8 will be modeled as repeated outcomes, with group, time, and the group × time interaction included as fixed effects and baseline MoCA score included as a covariate. The primary confirmatory comparisons will be the adjusted between-group differences at week 4 between the synchronous group and Control Group 1 and between the synchronous group and Control Group 2. To control the family-wise error rate for these two prespecified comparisons, the Holm adjustment will be applied.

The likelihood-based MMRM will constitute the primary approach to handling incomplete outcome data under the missing-at-random assumption, without requiring explicit imputation of missing outcome values. Multiple imputation by chained equations (MICE) will be performed as a sensitivity analysis to assess the robustness of the primary findings to the handling of missing data. Additional sensitivity analyses will further adjust for prespecified baseline variables, including baseline neurological severity and affected hemisphere. Descriptive within-group changes from baseline will also be reported; however, confirmatory conclusions regarding the primary outcome will be based on the prespecified between-group contrasts derived from the MMRM.

For other continuous variables, normality will be assessed using the Shapiro–Wilk test. Normally distributed data will be summarized as mean ± standard deviation, whereas non-normally distributed data will be presented as median and interquartile range [M (P25, P75)]. Appropriate parametric or non-parametric methods will be used for between-group comparisons according to data distribution. Categorical variables will be presented as frequencies and percentages and compared using the chi-square test or Fisher’s exact test, as appropriate. Ordinal variables will be analyzed using appropriate rank-based tests. All statistical tests will be two-sided, with *p* < 0.05 considered statistically significant unless otherwise specified for multiplicity-adjusted comparisons. Correlations between multimodal metrics will be examined using Pearson’s correlation coefficient for normally distributed data and Spearman’s rank correlation coefficient for non-normally distributed data.

#### 2.10.2 Multimodal data fusion

##### 2.10.2.1 Data preprocessing

MoCA and MMSE scale scores will be normalized to the [0, 1] interval to mitigate bias arising from disparate scoring ranges across assessment instruments. For neuroimaging and electrophysiological data, including TCD, the P300 component of ERP, and rs-fMRI, standardized preprocessing pipelines encompassing filtering, denoising, and feature extraction will be applied (32). Specifically, peak systolic velocity (*Vs*), end-diastolic velocity (Vd), and mean flow velocity (Vm) of the bilateral middle cerebral arteries will be extracted from TCD recordings; peak latency and amplitude of the P300 component will be quantified from ERP data; and core functional metrics including amplitude of low-frequency fluctuation (ALFF) and regional homogeneity (ReHo) will be computed from rs-fMRI data.

##### 2.10.2.2 Data fusion strategy

A feature-level fusion framework will be implemented to integrate validated features across all modalities, yielding an initial pool of approximately 250 candidate features (predominantly derived from regional rs-fMRI metrics, ERP parameters, and TCD hemodynamics). Given the limited sample size of the current cohort (*n* = 96) in contrast to this high-dimensional nature of the multimodal data, the application of machine learning algorithms (Support Vector Machine [SVM] and Random Forest [RF]) is strictly defined as an exploratory, hypothesis-generating analysis (33). The fused multimodal feature vector will serve as the input, with the magnitude of cognitive improvement as the output label. To stringently mitigate the high risk of overfitting, the entire analytical pipeline will be explicitly embedded within nested 5-fold cross-validation loops (34, 35). Specifically, to rigorously prevent data leakage, the methodology is operationalized as follows: (1) Missing data handling: Incomplete multimodal features will be imputed using Multiple Imputation by Chained Equations (MICE), with the imputation model fitted exclusively on the training folds and applied to the test folds. (2) Dimensionality reduction & Feature selection: Principal Component Analysis (PCA) will be applied for initial dimensionality reduction of the neuroimaging data, followed by Recursive Feature Elimination (RFE) to isolate the most predictive candidate features. Both steps will be executed strictly within the cross-validation training data. (3) Hyperparameter tuning: Model optimization via Grid Search will be conducted exclusively within the inner cross-validation loops. (4) Performance evaluation: Predictive accuracy for the regression task will be quantified on the hold-out outer test folds using Root Mean Square Error (RMSE) and Mean Absolute Error (MAE). Given the exploratory nature of this analysis, the primary objective of this modeling is not to develop or deploy a fully generalizable clinical prediction tool, but rather to preliminarily explore potential predictive multimodal features and assess the methodological feasibility of multi-source data fusion within this specific PSCI cohort.

## 3 Discussion

This study proposes an innovative rehabilitation protocol for PSCI using scalp acupuncture synchronized with taVNS based on the “central-peripheral-central” closed-loop rehabilitation theory. Through a randomized controlled trial design, this study aims to evaluate the potential therapeutic advantages of the synchronous intervention mode compared to the sequential intervention mode, while further exploring multimodal neurophysiological response patterns associated with intervention timing.

### 3.1 Logic of synchronous intervention guided by the “central-peripheral-central” closed-loop theory

PSCI is one of the most common and profoundly impactful complications following a stroke. The impairment of cognitive function not only directly affects the patient’s rehabilitation progress and quality of life but also serves as an independent risk factor for increased post-stroke mortality (36). However, current pharmacological treatments for PSCI have limited efficacy and are often accompanied by adverse reactions. Consequently, non-invasive neuromodulation techniques have emerged as a promising alternative or complementary strategies.

In recent years, the “central-peripheral-central” closed-loop rehabilitation theory has been widely validated and applied in the rehabilitation of post-stroke motor dysfunction. The core logic of this theory is that central interventions can directly activate functional brain regions and regulate neuroplasticity, while peripheral interventions drive central remodeling through peripheral sensory input. Together, they form a closed-loop effect of “bidirectional conduction and holistic rehabilitation.”

This study extends this theoretical framework to the temporal combination of scalp acupuncture and taVNS. Its innovation lies in the following: as a classic central intervention, scalp acupuncture acts directly on the functional representative areas of the cerebral cortex, improving cognition by regulating the functional connectivity of brain networks and neuroplasticity (37, 38). Meanwhile, taVNS stimulates the superficial branches of the vagus nerve in the concha, projecting through the nucleus tractus solitarii (NTS) to widespread brain regions such as the locus coeruleus and basal forebrain (14). This regulates neurotransmitter systems, including norepinephrine and acetylcholine, potentially facilitating central remodeling driven by peripheral stimulation (39). From a theoretical perspective, synchronous delivery may increase the temporal overlap between the central modulation associated with scalp acupuncture and the peripheral afferent input associated with taVNS. Whether this temporal overlap is associated with different clinical or neurophysiological outcomes remains to be determined in the present trial (40, 41).

### 3.2 Biological basis and scientific value of intervention timing

One of the core scientific questions of this study is whether the timing of scalp acupuncture and taVNS interventions affects therapeutic efficacy. This question touches upon a fundamental, yet under-explored, proposition in the field of neuromodulation: the temporal interaction between central and peripheral interventions.

From a neurobiological perspective, the reorganization of brain functional networks triggered by scalp acupuncture and the vagal-central afferent signals mediated by taVNS may involve different temporal dynamics. By activating vagal afferent fibers, taVNS can trigger the NTS-locus coeruleus-cortex projection pathway within tens of milliseconds to seconds, modulating the neuroexcitatory state of the whole brain (39). In contrast, the continuous needle retention of scalp acupuncture, combined with intermittent twirling and manipulating, may exert its effects through sustained improvements in cerebral blood flow and the upregulation of synaptic plasticity-related proteins (42, 43). Scalp acupuncture and taVNS may involve neurophysiological processes with different temporal characteristics (44). Therefore, synchronous and sequential administration may be associated with different patterns of neural and hemodynamic responses. Whether these timing differences translate into measurable differences in cognitive outcomes remains uncertain and will be examined in this trial.

### 3.3 Exploratory multimodal characterization of neurophysiological responses

Current clinical research on PSCI predominantly relies on neuropsychological scales as primary outcome measures. Although widely used, they are susceptible to confounding factors such as the subject’s education level, cooperation degree, and emotional state. They lack objective biological markers and struggle to elucidate the interventional pathways at a mechanistic level. This study develops a multimodal assessment system integrating behavioral, neuroimaging, neurophysiological, and hemodynamic measures, which provides substantial methodological value.

Specifically, the MoCA, MMSE, and MBI reflect improvements in cognitive function and activities of daily living at the clinical phenotypic level (45). rs-fMRI can reveal the functional remodeling of cognition-related circuits, such as the default mode network and executive control network, at the brain network level (46). The event-related potential P300, serving as an electrophysiological marker of cognitive processing, objectively reflects subclinical changes in attention and information processing speed (47, 48). TCD ultrasound reflects the potential modulatory effect of the intervention on intracranial blood flow velocity at the cerebral hemodynamic level (49). These four dimensions of indicators complement each other, providing a multidimensional view of the complete effect chain from brain function to behavior, thus overcoming the limitations of single-scale assessments (50).

Building on this, this study will apply feature-level fusion and machine-learning methods as exploratory, hypothesis-generating tools to identify candidate multimodal features associated with cognitive improvement and to characterize response patterns associated with intervention timing. This approach effectively integrates information from multi-source heterogeneous data to achieve exploratory predictive associations of cognitive rehabilitation outcomes. Simultaneously, it provides insights into the exploratory mechanistic associations of central–peripheral temporal modulation across multiple scales—from brain-network architecture through neuroelectric activity to hemodynamic responses—thereby catalyzing a paradigm shift toward a multidimensional, objective, and standardized framework for PSCI efficacy evaluation.

### 3.4 Limitations

This study has several limitations that should be acknowledged. First, it is a single-center clinical trial with a relatively homogeneous sample source, which may limit the generalizability of the findings; future multicenter studies are needed to further validate these results. Second, because scalp acupuncture and taVNS are inherently difficult to blind for both practitioners and participants due to the distinctive nature of the interventions, placebo effects and expectation bias may be introduced. Although these potential biases will be mitigated through assessor blinding, standardized operating procedures, and objective outcome measures, they cannot be completely eliminated. Third, because all three groups receive both active interventions, this design is specifically tailored to evaluate differences associated with intervention timing (synchronous versus sequential). It cannot establish the fundamental synergistic efficacy of the combination compared to either intervention alone. Additionally, the absence of a conventional sham control group inherently limits the ability to isolate true physiological effects from placebo responses or natural stroke recovery. Furthermore, the 4-week intervention period primarily captures short-term efficacy, making it difficult to evaluate long-term maintenance effects and improvements in long-term prognosis. Finally, the generalizability of the multimodal data fusion model still requires external validation in larger samples and multicenter settings.

## 4 Conclusion

In summary, this study will evaluate whether synchronous and sequential delivery of scalp acupuncture and taVNS are associated with different cognitive outcomes in patients with PSCI using a three-arm randomized controlled design. Multimodal assessments will be used on an exploratory basis to characterize neurophysiological response patterns associated with intervention timing. The study is not designed to establish the synergistic efficacy of the combined interventions or to provide definitive mechanistic or predictive conclusions. The findings are expected to provide preliminary evidence regarding intervention timing and inform future larger, multicenter studies.
